# Epidemiology of Primary Intracranial Tumors at a Tertiary Care Hospital in Central India: A Retrospective Study

**DOI:** 10.7759/cureus.88404

**Published:** 2025-07-21

**Authors:** Shiv S Mishra, Manish K Ahirwar, Siddhartha Nanda, Swaroopa M Rath

**Affiliations:** 1 Radiation Oncology, Paras HMRI Hospital, Patna, IND; 2 Radiation Oncology, Gandhi Medical College, Bhopal, IND; 3 Radiation Oncology, All India Institute of Medical Sciences, Raipur, IND

**Keywords:** astrocytoma, chhattisgarh, glioblastoma, india, primary brain tumors

## Abstract

Introduction: Primary intracranial tumors have become a significant public health concern in India in recent years. Due to a lack of awareness among the general population and poor healthcare facilities, patients often receive late diagnoses and suboptimal outcomes. This study aims to collect comprehensive data on the demographics and essential characteristics of brain tumor patients to provide a whole spectrum of clinical and histological data of primary intracranial tumors at a newly established radiotherapy department at the All India Institute of Medical Sciences (AIIMS), Raipur, India. The findings will help to develop better treatment strategies and programs to improve outcomes and management of primary intracranial tumors.

Methods: The study involved 196 patients diagnosed with primary intracranial tumors registered in the Department of Radiation Oncology between January 1, 2020, and December 31, 2023. Epidemiological data, including age, sex, and incidence rates of various intracranial malignancies classified according to the World Health Organization classification, were collected and analyzed between December 2023 and July 2024 to better understand the demographics and epidemiology of primary intracranial tumors.

Results: During the study period, the total number of cancer cases receiving radiotherapy at AIIMS, Raipur, increased 3.5 times in four years. The proportion of primary intracranial neoplasms to all cases presented to the Department of Radiation Oncology (AIIMS, Raipur) was 15.1% (36/237), 9.3% (47/503), 6.4% (51/786), and 7.6% (66/874), respectively, in the years 2020-2023. Overall, between 2020 and 2023, astrocytoma was the most common tumor, followed by glioblastoma, with incidences of 38% (61) and 27% (27), respectively. In the pediatric group, astrocytic tumors (29.4%; 10 cases), embryonal tumors (26.4%; nine cases), and craniopharyngioma (14.7%; five cases) were the most common presentations.

Conclusion: The increasing burden of primary intracranial tumors in India demands attention to improve access to healthcare and increase awareness about the early signs and symptoms of the disease. The data collected in this study can be used to develop effective strategies for managing and treating brain tumors in India.

## Introduction

It is important to note that the prevalence of brain tumors has been rising recently all over the world. Various factors, like an aging population and changes in environmental and lifestyle factors, contribute to this gradual rise. The recent advancement in diagnostic techniques and increased awareness among the general population have resulted in the diagnosis of a number of new cases. However, the etiology of brain tumors remains unknown, and more research is needed to understand it better.

The cancer burden is affecting developing countries like India much more than developed countries. Global Cancer Observatory 2022 data have revealed that the total number of new cancer cases is approximately 20 million, among which India contributes 1.4 million new cases, accounting for 7% of the global cancer burden [1]. Brain and central nervous system (CNS) tumors are ranked 19th and 14th globally and in India, respectively, as per their incidence. However, these are the 12th and 11th leading causes of death from cancer across the globe and in India, respectively. Brain tumors contribute to approximately 3.1% of all cancer incidences as well as mortality in India [1].

In India, one in 341 men has a cumulative risk of developing primary brain malignancy, while among women, one in 546 has the cumulative risk of developing primary brain malignancy [2].

According to the National Cancer Registry Programme (2012-2016), the estimated contribution of brain and CNS tumors to the total cancer burden in India in 2025 has been projected to be 2.3%, while it was 2.1% in 2016 [2].

In our study, we have intended to document patient demographics and tumor characteristics to provide complete clinical and pathological data on primary brain tumors treated at the newly opened radiation oncology department of the All India Institute of Medical Sciences (AIIMS), Raipur, India. The findings of this study will help develop better treatment strategies and programs to improve management and outcomes for patients with primary intracranial tumors.

## Materials and methods

This retrospective study was conducted at the Department of Radiotherapy, AIIMS, Raipur, between January 2020 and December 2023. We included 196 consecutively registered patients (both pediatric and adult) with radiologically (MRI/CT) or histopathologically confirmed primary intracranial tumors, while excluding those with metastatic brain lesions. Using a standardized data collection form, one researcher retrospectively extracted anonymized patient data from departmental medical records manually, including the following: 1) demographics (age, sex, and residence); 2) clinical presentation (symptoms, duration, and performance status); 3) diagnostic details (imaging findings, histopathology reports); and 4) treatment modalities (surgical intervention, radiotherapy protocols, and systemic therapy). The validated data were entered into a Microsoft Excel spreadsheet (Microsoft Corporation, Redmond, WA) with a predefined set of headings.

## Results

A total of 196 patients were included in the study, and all the relevant data were collected for the study. There was a clear male preponderance in the study group, with 59.7% (n = 117) male patients and 40.3% (n = 79) female patients.

**Figure 1:**
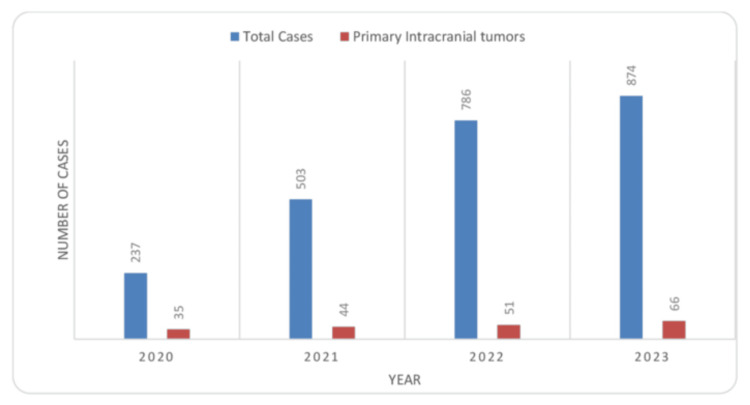
Yearwise distribution of all primary intracranial tumor cases and total number of cases noted each year

The total number of cancer cases receiving radiotherapy at our institute during the study period has increased 3.5-fold in three years, and the proportion of primary intracranial tumors to all cases presented to our department was 14.8% (35), 8.7% (44), 6.4% (51), and 7.5% (66) in the years 2020-2023 (Figure 1).

All the patients included in our study received multidisciplinary treatment according to the recommended treatment guidelines. One hundred seventy-nine patients (91.3%) underwent surgery, and in six patients (3.1%), stereotactic biopsy was done. However, 11 patients did not undergo any histopathological confirmation and were further managed based on their distinctive clinical and radiological features. All patients received radiotherapy in the form of definitive or adjuvant radiotherapy. Oral temozolomide and intravenous chemotherapy (such as vincristine; the bleomycin, etoposide, and cisplatin regimen; and bevacizumab) were also administered depending on the diagnosis and indications.

At our institute, we follow a histomolecular approach, incorporating a four-layered conclusion in the pathology report for CNS tumors. Histopathological diagnosis was aided by molecular markers like isocitrate dehydrogenase, Ki 67, P53, glial fibrillary acidic protein, synaptophysin, loss of the ATRX gene, S100, vimentin, epithelial membrane antigen, RELA fusion, Olig2, PanCK, STAT6, CD34, chromogranin, beta-catenin, MIB index, INI1, D2 40, ERG, PAS, OLIG2, BRAF600E, and 1p19q co-deletion, wherever applicable. It was observed that Ki67 had a range of less than 1%-30% in grade I and II tumors, and in grade III and IV tumors, Ki67 had a range of 5% to 75%.

Agewise distribution

**Figure 2:**
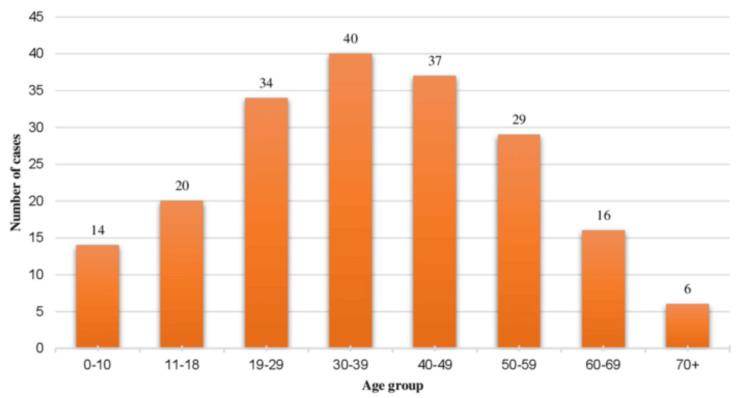
Agewise distribution of all primary intracranial tumor cases and number of cases in each age group (n = 196)

In our study, we found that the maximum proportion of intracranial tumors was diagnosed in the fourth decade; 54.8% of all patients were between the ages of 30 and 60 years; 34% of all the patients were younger than 30 years of age, whereas about 11.2% were aged 60 years and above (Figure 2).

Out of 196 patients, 28 (14.4%) were grade I, 52 (26.5%) were grade II, 30 (15.3%) were grade III, and 70 (35.7%) were grade IV. Sixteen (8.1%) patients were unable to be classified using the World Health Organization grading system.

**Figure 3:**
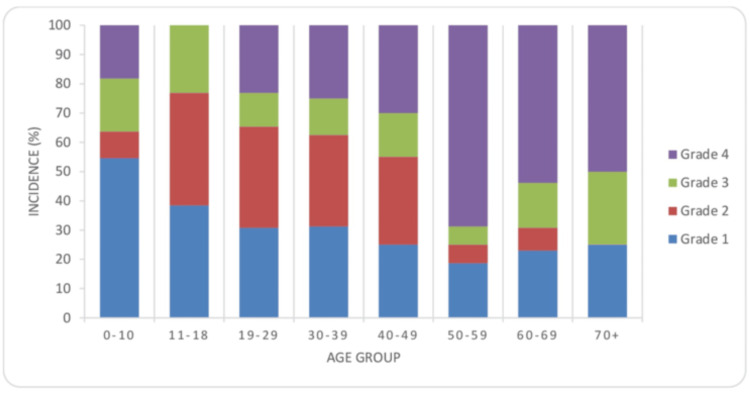
Agewise distribution of the World Health Organization tumor grades 1, 2, 3, and 4 depicted as percentage of total cases noted within each age group (n = 196)

We also observed that the incidence of grade I tumors was highest in the age group less than 10 years (54.54%), and there is a gradual decrease in the incidence of these tumors with increasing age. However, the incidence of grade IV tumors has increased from 2020 to 2023, following an increasing trend with age, with a maximum number of new cases in the over-50 age group (>50%). The proportion of grade II and III tumors remained variable throughout the study period (Figure 3).

Histopathological distribution

Overall, astrocytic tumors were the most common, followed by glioblastoma (GBM), with incidences of 36.2% and 22.4%, respectively. For ease of understanding, we have grouped the age groups into two categories: pediatric (0-18 years) and adult (19 years and above). The pediatric group comprised 17.3% (n = 34) of all patients, and the remaining patients were in the adult group (n = 162).

**Figure 4:**
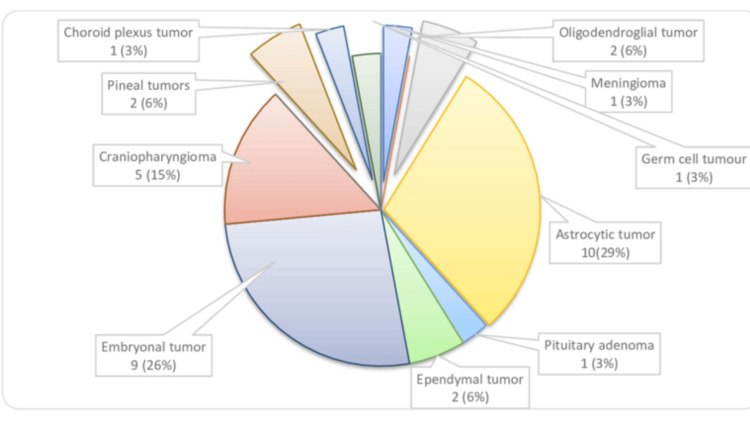
Distribution of primary intracranial tumors within the pediatric population (n = 34)

In the pediatric group, the most common histological presentation was astrocytic tumors, 10 (29.4%), followed by embryonal tumors, nine (26.4%), and craniopharyngioma, five (14.7%). All the embryonal tumors were medulloblastoma (Figure 4).

In adults, the most common histological presentation was astrocytic tumors (37.6%), followed by GBM (27.2%), oligodendroglioma (12%), meningioma (7%), pituitary adenoma (6%), and craniopharyngioma (4%). The remaining 6.2% of the patients had embryonal tumors, oligoastrocytomas, pineal tumors, ependymal tumors, primary CNS lymphomas, and germ cell tumors. Out of all the patients in the adult age group, three had gliosarcomas, a rare histopathologic variant of GBM.

Addiction and comorbidities

Eighty percent of patients did not have a history of any form of addiction (smoking, tobacco, or alcohol), and approximately 76% of patients neither had any comorbidities like diabetes or hypertension nor were on any medications.

Comparison with Indian and international data

Our data were very similar to previously published Indian data by Jaiswal et al. [3] and Jalali and Datta [4] with respect to incidence and median age for all the histologies of brain tumors (Table 1).

**Table 1:** Median age at presentation for various histological subtypes and comparison with national and international data NA: not available

Diagnosis	Number (n = 196)	Pediatric (≤18 years) (n = 34)	Adult (≥18 years) (n = 162)	Median age of presentation			
				Tata Memorial Hospital(2006) [4]	National Institute of Mental Health and Neurosciences(2010-2014) [3]	Central Brain Tumor Registry of the United States(2015-2019) [5]	All India Institute of Medical Sciences, Raipur (2020-2022) (present study)
Astrocytoma							
Pilocytic	3	1	2	16	14	11	14
Anaplastic	10	1	9	36	36	52	42
Glioblastoma	40	0	40	50	50	65	52
Diffuse astrocytoma	20	3	17	NA	37	45	34
Oligodendroglial tumors	9	1	8	37	40	44	40
Oligoastrocytic tumors	1	0	1	NA	36	45	32
Pituitary adenoma	9	1	8	39	42	51	37
Craniopharyngioma	11	4	7	20	22	45	24
Meningioma	9	1	8	46.5	45	67	45
Ependymal tumors	3	2	1	18.5	18	45	15
Pineal tumors	1	1	0	18.5	25.5	34	13
Lymphomas	1	0	1	NA	50	67	73
Embryonal tumors	10	7	3	NA	8	8	9.5
Germ cell tumors	1	0	1	NA	16	15	13
Arteriovenous malformations	1	0	1	21	NA	50	27
Choroid plexus tumors	1	1	0	NA	13	20	8
Nerve sheath tumor	4	0	4	NA	40	58	NA

However, comparison with the Central Brain Tumor Registry of the United States (CBTRUS) data revealed that the median age of diagnosis in our study was lower in most of the histologies except pilocytic astrocytoma, lymphoma, and embryonal tumors [5]. The median age of diagnosis for the malignant tumors was almost a decade earlier in our study than in the CBTRUS data. There was a huge difference of approximately 20 years in the median age of diagnosis of benign tumors between our study and CBTRUS data. However, the age of diagnosis was found to be similar to Indian data.

## Discussion

In India, brain tumors are a growing health concern, and their incidence has been increasing over the years [2]. According to available data, brain tumors account for approximately 1%-2% of all cancers in India and affect people of all ages. However, they are more commonly seen in people over 50 years of age.

Brain tumor cases in India often go undiagnosed until the later stages due to limited healthcare access and less awareness, resulting in poor prognosis and lower survival rates. Our research indicates that a significant percentage of primary intracranial tumors were diagnosed during the third decade of life, which is consistent with findings reported by Jaiswal et al. [3] and Jalali and Datta [4]. Additionally, Jaiswal et al. found that glioma patients were diagnosed at a younger age compared to individuals with other types of primary brain tumors. Middle-aged adults, between the ages of 30 and 60, accounted for half of all primary intracranial tumor cases, indicating that they are at higher risk of developing such tumors. Although Jaiswal et al. did not report the age distribution of patients with primary brain tumors in their study, our research revealed a distinct age distribution pattern. Specifically, our findings indicate that more than 50% of primary intracranial tumor cases occurred in individuals aged between 30 and 60 years, 34% in patients younger than 30 years, and 11.2% in patients aged 60 years and above. This deviation highlights the importance of conducting studies that provide detailed information about age distribution in addition to the relative frequency of different types of primary brain tumors, as these factors can significantly impact the diagnosis, treatment, and prognosis of patients with intracranial tumors. This finding is consistent with the study by Jaiswal et al. [3], which also reported a high incidence of primary intracranial tumors in pediatric and young adult populations. The age distribution of brain and CNS tumors reported by Ostrom et al. [5] and Manoharan et al. [6] differs slightly from our study. Their research revealed that individuals between 50 and 59 years old had the highest incidence of these tumors, followed by those in the 40-49 age group. These differences in age distribution could be due to variations in study design, patient populations, and geographical locations. It is crucial to note that these variations can significantly impact the interpretation and generalizability of study findings.

In a study by Jain et al. [7], astrocytoma was the most common histological type of pediatric brain tumor, accounting for 34.7% of cases. Medulloblastoma followed at 22.7%, craniopharyngioma at 10.2%, and ependymoma at 9.8%. There were variations in the relative proportions of tumor types across the six different centers, with medulloblastoma being the most common in three centers and astrocytoma in the other three. In our study, the pediatric group comprised 17.3% (n = 34) of all patients, with astrocytic tumors being the most common histological presentation (29.4%), followed by embryonal tumors (26.4%) and craniopharyngioma (14.7%).

It is worth noting that only 11.2% of the patients diagnosed with intracranial tumors in our study were aged 60 years or above, which is lower than the proportion of patients in the 30-60-year age group. However, it is important to consider that this finding may have been influenced by factors such as variations in healthcare accessibility for different age groups.

The observation in our study that grade I tumors were most frequently diagnosed in patients aged less than 10 years is consistent with previous research reporting a higher incidence of benign tumors in pediatric age groups [8,9].

In our study, astrocytic tumors (29.4%), embryonal tumors (26.4%), and craniopharyngioma (14.7%) were the most common histological presentations in the pediatric age group. In contrast, astrocytic tumors (37.6%) and GBM (27.2%) were the most common histological presentations in adults [10,11]. In contrast, Jaiswal et al. [3] reported that the most common primary intracranial tumors were meningioma (27.2%), followed by gliomas (25.3%) and pituitary adenomas (14.3%), while Manoharan et al. [6] found that the most common primary brain and CNS tumors were meningioma (25.2%), followed by pituitary tumors (16.4%) and gliomas (14.4%).

Our study reported a male preponderance of 59.7% among the patients, which is similar to the finding of approximately 60% male patients in the study by Jalali and Datta [4]. However, the proportion of male patients in our study was slightly higher than those reported by Jaiswal et al. [3] (53.8% male patients) and Manoharan et al. [6] (54.6% male patients).

Our study has several limitations that should be acknowledged. First, the relatively small sample size (n = 196) from a single tertiary care institution may limit the generalizability of our findings to the broader population of central India. Second, as our comparative reference data did not include confidence intervals, we were unable to calculate these statistical measures for our dataset; hence, it is purely a descriptive study with no analytical analysis. Third, being a newly established cancer treatment facility, there may have been limited patient awareness about available treatment options, potentially introducing selection bias. These factors suggest that our results should be interpreted with caution. Future multicenter studies with larger, more diverse patient populations across different regions of India are needed to validate our findings.

## Conclusions

Brain tumors are a serious but often overlooked health issue in India. While less common than other cancers, they affect both children and adults, with late detection worsening outcomes due to limited awareness and healthcare access.

Our study provides key data on primary brain tumors in Chhattisgarh, which can help shape better treatment strategies and healthcare programs. This data can serve as a foundation for developing targeted treatment strategies and region-specific healthcare programs to improve early detection, optimize therapeutic approaches, and enhance overall treatment outcomes.
